# Social Determinants of Health Inequities in Indigenous Canadians Through a Life Course Approach to Colonialism and the Residential School System

**DOI:** 10.1089/heq.2019.0041

**Published:** 2019-07-25

**Authors:** Paul J. Kim

**Affiliations:** ^1^ Department of Medicine, University of Toronto, Toronto, Canada.; ^2^ Department of Biochemistry and Microbiology, University of Victoria, Victoria, Canada.

**Keywords:** Aboriginal, Canada, colonialism, health equity, social determinants of health

## Abstract

Indigenous populations in Canada have experienced social, economic, and political disadvantages through colonialism. The policies implemented to assimilate Aboriginal peoples have dissolved cultural continuity and unfavorably shaped their health outcomes. As a result, indigenous Canadians face health inequities such as chronic illness, food insecurity, and mental health crises. In 2015, the Canadian government affirmed their responsibility for indigenous inequalities following a historic report by the Truth and Reconciliation Commission of Canada. It has outlined intergenerational traumata imposed upon Aboriginals through decades of systemic discrimination in the form of the Residential School System and the Indian Act. As these policies have crossed multiple lifespans and generations, societal conceptualization of indigenous health inequities must include social determinants of health (SDOH) intersecting with the life course approach to health development to fully capture the causes of intergenerational maintenance of poor health outcomes. To provide culturally sensitive care for those who have experienced intergenerational trauma, health care providers should be aware of and understand two key SDOH inequity influencing the indigenous life course, including the residential school system and loss of socioeconomic status, over time due to colonialism.

## Introduction

Compared to the general population, indigenous Canadians suffer from disproportionate increases in diabetes, hypertension, substance abuse, mental health concerns, and overall morbidity and mortality in addition to having significantly reduced life expectancy.^1,2^ To conceptualize the origins of these disparities, exploring the social determinants of health (SDOH) can provide a strong framework of understanding. Proximal SDOH are often relied upon to explain inequities on a social scale as they include direct health-related behaviors, physical environments, and socioeconomic status (SES).^3–5^ Systems level influencers such as the health care system, education system, community, environmental stewardship, and cultural continuity have been deemed intermediate SDOH, while colonialism, racism, and self determination have been identified as distal SDOH.^3,5^ Focus will be given to the most pertinent distal SDOH for indigenous Canadians, colonialism, and how it has shifted the trajectories of the intermediate and proximal SDOH to produce intergenerational health inequities.

For Aboriginal populations, health is an overarching concept through the stages of life that comprise physical and mental wellbeing, as well as spiritual and cultural cohesion. Thus, effective indigenous health policies must be culturally sensitive and not impose definitions of health from ethnocentric perspectives.^6^ By definition, colonial policies contained culturally insensitive agendas to promote assimilation. The residential school system promoted family separation and cultural dissociation during childhood and adolescence, critical stages in the life course approach to health development (LCHD) that determine cognitive and social capacity into early adulthood and beyond.^7^ By utilizing the LCHD, a theory that traces health problems back to specific stages of life and their associated SDOH, it is possible to understand how cumulative disadvantages imposed upon Aboriginals have not only caused health inequities but also intergenerational trauma.^8,9^

## Residential School System

The residential school system was a highly controversial policy which instigated the dissociation of spiritual and cultural cohesion within Aboriginal communities.^5^ It was one of the most compelling pieces of evidence illustrating the nature of colonialism and its deleterious policies that caused the decline of indigenous populations in not only their overall health but also their sociocultural functioning. When the Indian Act was passed in 1876, this provided the foundation for the residential school system until 1996 when the last federal residential school was closed.^10^ The Canadian government mandated this policy to facilitate the assimilation of Aboriginal children into Euro-Canadian culture.^5^ Childhood and adolescence are vital times of cognitive learning and relationship maturation, so it is important to consider the impact of the residential school system on indigenous health with respect to the LCHD. In childhood, access to quality education is a protective factor for future health status because it promotes academic and social skills development, increases health literacy, and enables positive interpersonal development.^11^ However, this is only possible if the educational setting is provisioned with caring instructors, support networks, and ample resources to maintain a rich learning environment. The residential school system was not designed to provide any of these essential resources. Children were taken from their families to attend residential schools by government officials, placing extreme emotional stress on indigenous parents and their children.^10^ Being forced to attend against their will, over 81.3% of students felt isolated from their families.^5^ Family separation in childhood has been shown to be a major risk factor for mental disorders such as depression.^12^ Indeed, the literature has shown that family stress can cause dysregulation in brain function later in life.^13^ These findings demonstrate how family disruption may have negatively influenced Aboriginal mental health at later stages in the LCHD due to colonial policies. In school, ethnocentric rules were mandated and children were banned from speaking their native language or practicing any cultural customs.^10^ A study found that 76.8% of students recognized a significant loss in cultural identity due to their residential school experience.^5^ While our society may not include cultural identity in the definition of health, Aboriginals consider cultural cohesion to be strongly related to their health.^5^ Therefore, loss in cultural identity was a loss of health due to the residential school system instigated through colonial perspectives. Instructors enforcing these policies further contributed to the problem by verbally, emotionally, and even physically abusing students.^5^ As a child, negative relationships with caregivers and teachers can have serious implications on behavior, adjustment, and attitudes in adolescence and early adulthood.^14^ Childhood abuse has also been linked to poor health status, leading to increased hospitalizations for physical and psychological illnesses in adulthood.^15^ In a 2012 study by Elias et al.,^16^ 48.1% of residential school attendees reported an abuse history, and those who were abused experienced increased rates of suicidal thoughts and suicide attempts. An important aspect of the child-teacher relationship is closeness, which is positively correlated with success in school and personal initiative.^14^ The abuse experienced by these children eliminated any possible positive outcomes related to academic success and ambition that residential schools were supposed to promote which is evident today by more than half of the Canadian Aboriginal population without high school diplomas.^17^ For students who managed to graduate, ineffective education resulting from trauma, cultural inhibition, and racism by teachers led to reduced employment opportunities and decreased SES.^18^ Clearly, the residential school system was detrimental to the indigenous population and paved the way for dysfunctional social and cognitive development in countless generations of Aboriginal children even after residential school policies were removed. The LCHD views health on a continuum, and as such, the survivors of the residential school system were not the only ones affected. Attendees who experienced trauma may exhibit not only intragenerational reduced health status but they also pass on their competencies or perhaps, lack of competencies, to future generations. Being in an indigenous family with residential school history has been linked to increased stress and decreased overall wellbeing, supporting the finding that colonialism has resulted in cumulative health disadvantage for existing generations of Aboriginals even if they did not directly live through colonial policies.^19^ Nonattendees of parents who were residential school survivors experience increased abuse which appears to solidify the concept of intergenerational trauma.^16^ Personal interviews revealed that the attendees felt that their parenting abilities were inadequate due to the lack of practical education and overall trauma they lived through.^20^ These individuals who did not gain key competencies and developed negative relationships in their childhood and adolescence at residential schools were likely to reproduce such environments for their offspring, causing a vicious cycle of health disadvantage.^20^ This meant that their children would also be less able to cope with stressors in life, leading to negative health outcomes.

## Loss of SES

The residential school system ultimately limited employment opportunities for Aboriginals, which led to reduced SES.^18^ This was exacerbated by the lack of economic development in indigenous communities because the Canadian government implemented economic policies that focused on development in new or unseated lands from Aboriginals.^5^ The reserve system was one of the colonial policies that mediated economic isolation in indigenous communities. By placing Aboriginals on reserves and providing them with minimal financial support and no sustainable economic opportunities, the government forced them to integrate into new settler communities or succumb to relative poverty. Today, we know that many people on reserves suffer from impoverished living conditions and report that the lack of resources contribute to their economic insecurity.^5^ SES has been strongly correlated with health outcomes which is known as the socioeconomic gradient in health.^4^ People with lower SES tend to have reduced health status compared to their higher SES counterparts.^4^ This is a global phenomenon and the indigenous population is no exception. The unemployment rate is over two times higher for Aboriginal people relative to the general population.^5^ Unemployment has been linked to increased physical and mental illness and mortality, further supporting low SES as a risk factor for poor Aboriginal health.^21^ The health consequences of poor economic development and SES in indigenous communities are aggravated by the lack of access to basic goods and services such as healthy foods and medical support. A significant number of Aboriginals cannot afford medical treatment or transportation to hospitals, illustrating the severity of relative poverty being experienced on reserves.^22^ Their ability to access appropriate medical attention is limited by their low SES, which is a direct causative relationship to the poor health seen in Aboriginal communities across the nation. Furthermore, the lack of healthy food options act in synergy with low SES. For example, women with low income tend to be more obese than women with high income, which is intensified when Aboriginals cannot access any healthy foods.^23^ Children are more likely to be obese if their parents are obese, indicating that behaviors related to health are learned directly from parents.^24^ As obesity is associated with morbidity and increased risk for disease, this poses evidence for negative health outcomes later in life due to low SES impacting early childhood behavioral development. With the prevalence of food insecurity caused by low SES in Aboriginal families, it is possible to conclude that poor diet plays a key role in the health inequities seen on reserves. Moreover, nutrition is tightly tied to academic success, and children with low SES are less likely to succeed in school resulting in higher unemployment.^25^ Therefore, indigenous children from low SES families may not be able to work to their full potential in school due to inadequate nutrition, leading to health disadvantage later in life. Poor nutrition is a cumulative process because the body relies heavily on the food resources available during childhood and adolescence to determine physical abilities, cognition, and health outcomes in adulthood and beyond. With a compromised diet, as seen in many indigenous communities with low SES, children can experience poor physical and mental growth and increased susceptibility to disease in adulthood.^26^

## Conclusion

The effects of colonialism on the Canadian Aboriginal population are far-reaching and evident in the health inequities seen today. The LCHD can be used to illustrate the impact of colonial policies on existing and future generations because it provides a platform to study the SDOH through the LCHD within the Aboriginal community and helps us define indigenous health in a culturally sensitive manner. While there have been many detrimental policies such as the residential school system and reserve system, we can study how the intentions and implementations of these policies can be changed for positive outcomes. Although the intergenerational impact of colonialism on Aboriginals has manifested itself in negative health outcomes, affirmative action can be taken to break the cycle of cumulative health disadvantages. Understanding and integrating historical knowledge into trauma informed care will also prove beneficial for this population and improve relationships between health care providers and indigenous patients. We can ensure that health at each life stage, particularly at sensitive stages such as childhood and adolescence, is addressed according to the needs of Aboriginals by directly working together with indigenous patients and communities.

## Abbreviations Used

LCHD: life course approach to health development
SDOH: social determinants of health
SES: socioeconomic status
